# Book Reviews

**Published:** 1900-10

**Authors:** 


### *BOOK REVIEWS, PAMPHLETS, EXCHANGES.*

### *BOOK REVIEWS.*

[Contributions solicited for review.]

PRACTICAL URANALYSIS AND URINARY DIAGNOSIS. A Manual for the Use of Physicians, Surgeons and Students. By Charles W. Purdy, LL.D., M.D., Queens University, Fellow of the Royal College of Physicians and Surgeons, Kingston, Canada; Professor of Clinical Medicine at the Chicago Post-Graduate Medical School; Author of "Bright's Disease and Allied Affections of the Kidneys;" also of "Diabetis: Its Causes, Symptoms and Treatment." Fifth Revised and Enlarged Edition. With numerous Illustrations, including Photo-engravings, Colored Plates and Tables for estimating total solids from Specific Gravity, Chlorides, Phosphates, Sulphates, Albumen, Reaction of Proteids, Sugar, etc., etc., in Urine. 6x9 inches. Pages xvi.—406. Extra Cloth, $3.00 net. F. A. Davis Company, Publishers, 1914–16 Cherry street, Philadelphia.

The new edition of this well known work contains much new material and original matter. The subject of diagnosis by the urine is thoroughly considered. There is no better work on this important subject than this.

---

THOMPSON'S PRACTICAL MEDICINE. A Text-Book of Practical Medicine. By William Gilman Thompson, M.D., Professor of Medicine in Cornell University Medical College, New York City, Physician to the Presbyterian and Bellevue Hospitals, New York. In one magnificent octavo volume of 1,010 pages, with 79 engravings. Cloth, $5 net; leather, $6 net; half morocco, $6.50 net. Lea Brothers & Co., publishers.

In this day of books when publishers and authors vie with each other for the completeness and excellence of their productions, it is difficult to award the palm. Thompson's work has many points to commend it. The full and ripe experience of the author, his standpoint that the main end of medicine is to prevent and to cure disease, his position being an authoritative one and leading him to understand the needs of a student and to give him what he wants, all these unite to bring the book to a high plane. It will prove popular, beyond doubt.

---

A MANUAL OF PERSONAL HYGIENE: Edited by Walter L. Pyle, A.M, M.D., Assistant Surgeon to Wills Eye Hospital, Philadelphia; Fellow of the American Academy of Medicine; former Editor of the *International Medical Magazine*, etc. Contributors: J. W. Courtney, M.D., Walter L. Pyle, M.D., George Howard Fox, M.D., B. Alexander Randall, M.D., E. Fletcher Ingalls, M.D., G. N. Stewart, M.D., and Charles G. Stockton, M.D. Illustrated. Philadelphia: W. B. Saunders & Company. 1900. Price, cloth, $1.50 net.

This small book presents in a practical way many things that a physician will find useful, not only to know but to practice. Personal hygiene is more often neglected than observed, to the lasting detriment of good health. This work teaches right living and its precepts should be carried out.

---

THE ANATOMY OF THE BRAIN. A Text-Book for Medical Students. By Richard H. Whitehead, M.D., Professor of Anatomy in the University of North Carolina. Illustrated with forty-one engravings. 6¼x9¼ inches. Pages, v–96. Extra Vellum cloth, $1.00, net. The F. A. Davis Co., Publishers, 1914–16 Cherry street, Philadelphia, Pa.

This work will be found to simplify greatly the anatomy of the brain. It is concise, practical and clear, and tends to make plain much that is likely to confuse a student in the rather complicated exposition of the subject in the large works on anatomy.

---

POLK'S MEDICAL REGISTER OF THE UNITED STATES AND CANADA FOR 1900.

This edition seems to contain absolutely every kind of information of interest to or concerning the physicians of the United

States, Canada and our new possessions. Other editions have been very complete, but this covers many points not touched in the others. It is a regular *vade mecum.*

---

THE TREATMENT OF FRACTURES. By Charles Locke Scudder, M.D., Surgeon to the Massachusetts General Hospital, Out Patient Department; Assistant in Clinical and Operative Surgery in the Harvard Medical School; assisted by Frederic J. Cotton, M.D. With 585 illustrations. Philadelphia: W. B. Saunders, 925 Walnut street, 1900. Price, $4.50.

This work is on a subject of interest to the general practitioner as well as to the surgeon. A knowledge of it may save one from much trouble and litigation, as nothing is more liable to show ignorance or inexpertness on the part of the medical attendant than results of the treatment of fracture. The book endeavors to simplify diagnosis and to direct the proper treatment. It is fully illustrated and is highly practical.

---

IN the "Institute Etchings" in the *Journal of Obstetrics*, Dr. C. B. Kinyon states he has found Dr. Skene's method for dispersing gas accumulation in the abdomen is efficacious. This is the method: " He takes about an ounce of a solution of gum acacia, and in this he puts eight or ten grains of quinine that has been made perfectly soluble by sulphuric acid, and puts this mixture into as much water as he wants to make it warm—probably about four ounces of water—injects this into the rectum. What does this do? In scores of cases for me it has done this: where the abdomen was distented and the patient was unhappy and uncomfortable, the gas would pass off in enormous quantities; then as soon as the over-distention was controlled the bowels would contract, the paralysis pass off and often there would be a rapid recovery. I have seen the temperature drop four degrees in six hours after the use of this quinine enema."

